# Effects of vitamin E supplementation on renal non-enzymatic antioxidants in young rats submitted to exhaustive exercise stress

**DOI:** 10.1186/1472-6882-11-133

**Published:** 2011-12-20

**Authors:** Sérvio A Bucioli, Luiz Carlos de Abreu, Vitor E Valenti, Claudio Leone, Helio Vannucchi

**Affiliations:** 1 Laboratório de Química e Bioquímica de Alimentos, Universidade de São Paulo (USP), São Paulo, SP, Brasil; 2 Departamento de Morfologia e Fisiologia, Faculdade de Medicina do ABC, Santo André, SP, Brasil; 3 Departamento de Fonoaudiologia, Faculdade de Filosofia e Ciências, Universidade Estadual Paulista, UNESP, Marília, SP, Brasil; 4 Departamento de Saúde Materno-infantil, Faculdade de Saúde Pública, Universidade de São Paulo, São Paulo, Brasil

## Abstract

**Background:**

Exercise stress was shown to increase oxidative stress in rats. It lacks reports of increased protection afforded by dietary antioxidant supplements against ROS production during exercise stress. We evaluated the effects of vitamin E supplementation on renal non-enzymatic antioxidants in young rats submitted to exhaustive exercise stress.

**Methods:**

Wistar rats were divided into three groups: 1) control group; 2) exercise stress group and; 3) exercise stress + Vitamin E group. Rats from the group 3 were treated with gavage administration of 1 mL of Vitamin E (5 mg/kg) for seven consecutive days. Animals from groups 2 and 3 were submitted to a bout of swimming exhaustive exercise stress. Kidney samples were analyzed for Thiobarbituric Acid Reactive Substances to (TBARS) by malondialdehyde (MDA), reduced glutathione (GSH) and vitamin-E levels.

**Results:**

The group treated with vitamin E and submitted to exercise stress presented the lowest levels of renal MDA (1: 0.16+0.02 mmmol/mgprot vs. 2: 0.34+0.07 mmmol/mgprot vs. 3: 0.1+0.01 mmmol/mgprot; p < 0.0001), the highest levels of renal GSH (1: 23+4 μmol/gprot vs. 2: 23+2 μmol/gprot vs. 3: 58+9 μmol/gprot; p < 0.0001) and the highest levels of renal vitamin E (1: 24+6 μM/gtissue vs. 2: 28+2 μM/gtissue vs. 3: 43+4 μM/gtissue; p < 0.001).

**Conclusion:**

Vitamin E supplementation improved non-enzymatic antioxidant activity in young rats submitted to exhaustive exercise stress.

## Background

During exercise stress, free radicals may be produced in excess of the body's natural defense. Strenuous exercise increases the whole body and tissue oxygen consumption up to 20 fold, which then elevates electron leakage from the mitochondrial transport system and disturbs the intracellular pro-oxidant and antioxidant homeostasis [1]. There have been many reports showing that exercise causes oxidative stress, e.g., the direct detection of free radical generation in rat skeletal muscle [2] and kidney [3] increases in oxidative damage biomarkers such as thiobarbituric acid reactive substances.

The protective capacity of the antioxidant defense system of sedentary individuals may therefore be more easily exceeded under conditions of acute physical exercise. Exogenous antioxidants, primarily obtained as nutrients or nutritional supplements, may help to counteract the oxidative stress of exercise in such subjects unaccustomed to physical activity. Supplementations are a non-pharmacological therapy that has been received attention in the literature [4-7]. More than 40% of people who practice physical activity use some type of dietetic supplementation in order to keep a good health. Vitamin E (a-tocopherol) seems to be a very important agent in providing protection against oxidation of cellular lipids by free radicals that are potentially damaging byproducts of cellular metabolism [8]. Vitamin E supplementation was shown to present protective effects against deterioration of kidney function in rats with streptozotocin-induced Type 1 diabetes mellitus [9].

Exercise stress was already shown to increase oxidative stress in old [3] and just weaned rats [10]. However, reports of increased protection afforded by dietary antioxidant supplements against ROS production during exercise are conflicting. For instance, several authors observed little or no effect of vitamin C and E supplementation on plasma ascorbic acid [11] and lipid peroxidation (LP) [11-13] levels whereas others have demonstrated increases in the concentration of ascorbic acid [14] and unchanged LP level [15] in blood following supplementation. Thus, we aimed to evaluate the effects of vitamin E supplementation on renal non-enzymatic antioxidants in young rats submitted to exhaustive exercise stress.

## Methods

### Animals

Thirty-two just weaned male Wistar rats weighing an average of 95.5 g were used. The animals were bred at the Central Animal House of the Ribeirão Preto Campus of the University of São Paulo. The animals were kept in plastic cages (a maximum of 4 rats per cage) in a room with controlled temperature (24-28°C) and luminosity (a 12 h light:12 h dark cycle) with free access to water and balanced food rations. The animals were kept in accordance to the guidelines of the Committee on Care and Use of Laboratory Animals of the National Research Council of the National Institutes of Health. All procedures were approved by the Ethics Committee in Research of The Faculdade de Medicina de Ribeirão Preto da Universidade de São Paulo (protocol 003/08).

### Experimental Design

Animals were randomized into three groups: 1) the control group (n = 8), in which rats were treated with gavage administration of 1 mL of water for seven consecutive days; 2) exercise stress group (n = 8), in which rats were treated with gavage administration of 1 mL of water for seven consecutive days and submitted to exhaustive exercise stress and; 3) exercise stress + Vitamin E group (n = 8), in which rats were treated with gavage administration of 1 mL of Vitamin E (5 mg/kg, gamma tocopherol) for seven consecutive days and submitted to exhaustive exercise stress. This period of treatment is able to achieve significant changes in antioxidant levels [10].

Groups submitted to exhaustive exercise stress were submitted to one bout of swimming exercise stress until exhaustion at the last day of the experiment (7^th^). They were submitted to exercise consisting of swimming in a glass tank (100 cm long, 50 cm wide, 80 cm deep) containing water and maintained at 32°C, when the animal stop swimming it was removed from the water, since it corresponds to a exhaustive situation [16]. The depth of the tank prevented the animals from resting their tails on the bottom of the tank while swimming. Swimming was selected because muscle trauma caused by prolonged running, exercise-stimulated electric shock, and plyometric contractions could be avoided. These factors alone could induce oxidative stress [17].

### Laboratory Methods

After swimming, the animals were killed by ether inhalation and fragments of the kidney were removed for determination of malondialdehyde (MDA), reduced glutathione (GSH), and vitamin E concentrations. Concentration of MDA, an indirect product of lipid peroxidation, was determined by the thiobarbituric acid reactive substance (TBARS) test, a technique commonly used in lipoperoxidation studies [18]. Acid-soluble thiols were quantified to determine tissue GSH levels [19]. Vitamin E (a-tocopherol) was determined by high-performance liquid chromatography (HPLC) on a C-18 column (Shimpack CLC-ODS 4.6 cm, 25 cm) and a 4 mm, 1 cm pre-column at a flow of 2.0 mL/min [20].

### Statistical Analysis

Data are reported as mean ± standard error of the mean (SEM). Statistical significance was assessed by analysis of variance (ANOVA), followed by Dunn post hoc test. Differences were considered significant when the probability of a Type I error was lower than 5% (p < 0.05).

## Results

**Figure 1:**
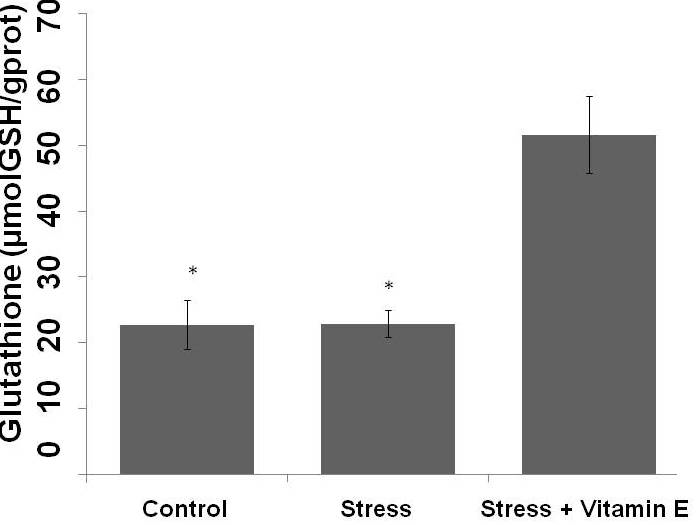
**Concentrations of renal reduced glutathione (GSH) in control (Control), exercise stress (Stress) and exercise stress treated (Stress + Vitamin E) groups**. *p < 0.0001: Different from Stress + Vitamin E group.

We observed that GSH concentrations in the kidney were elevated in the group submitted to exercise stress treated with Vitamin E compared to control and exercise stress not treated groups (Figure 1). This finding indicates that Vitamin E treatment increased renal GHS levels in rats submitted to exercise stress.

**Figure 2:**
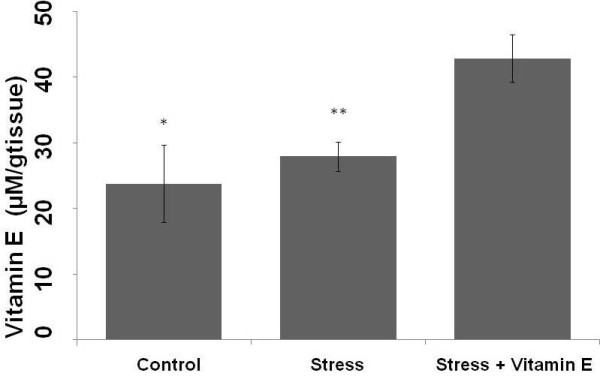
**Concentrations of renal Vitamin E in control (Control), exercise stress (Stress) and exercise stress treated (Stress + Vitamin E) groups**. *p < 0.001: Different of Stress + Vitamin E group. **p < 0.005: Different from Stress + Vitamin E group.

Moreover, in the group submitted to exercise stress treated with Vitamin E renal Vitamin E concentration was also higher than the control and exercise stress groups (Figure 2). It supports the fact that Vitamin E supplementation increased non enzymatic antioxidants activity in the kidney of rats submitted to exercise stress.

**Figure 3:**
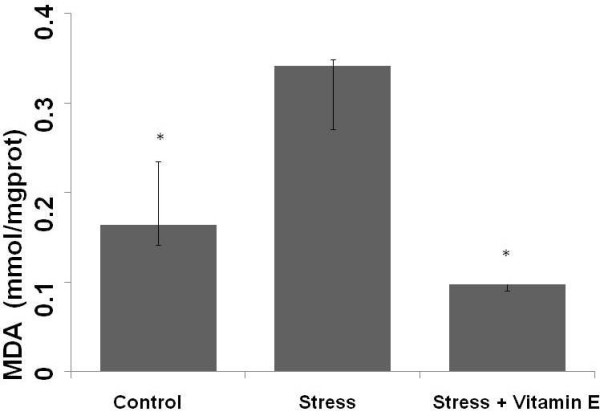
**Concentrations of renal malondialdehyde (MDA) in control (Control), exercise stress (Stress) and exercise stress treated (Stress + Vitamin E) groups**. *p < 0.0001: Different from Stress group.

In order to evaluate the renal oxidative stress, we evaluated the concentration of MDA, an indirect product of lipid peroxidation. Figure 3 indicates that MDA was higher in the group submitted to exhaustive exercise stress compared to the group submitted to exercise stress treated with Vitamin E and control group. This result indicates that Vitamin E treatment attenuated the increase in reactive oxygen species production caused by exhaustive exercise stress.

## Discussion

In this investigation we endeavored to evaluate the effects of Vitamin E supplementation by gavage on MDA, glutathione and Vitamin E levels in the kidney of young rats submitted to exhaustive exercise stress. As a main finding, Vitamin E pretreatment attenuated the responses caused by exhaustive exercise stress regarding oxidative stress, because it decreased the production of MDA in the kidney. In addition, Vitamin E pretreatment increased renal Vitamin E and GSH levels. We decided to use Vitamin E supplementation, because previous studies suggested that more than 40% of people from different ages who practice physical activity use some type of dietetic supplementation in order to keep a good health. Among the most consumed supplementations, we may include antioxidants such as Vitamin E [8].

### Effects of vitamin E on renal oxidative stress induced by exercise stress

The effects of vitamin E on renal oxidative stress after exhaustive exercise stress were not yet investigated in the literature. Our findings indicate that Vitamine E supplementation decreased renal oxidative stress caused by exhaustive exercise stress, because it reduced renal MDA levels in the group treated with Vitamin E submitted to exercise stress. Vitamins, as well as minerals and trace elements, have become of great interest in the world of sports because of their supposed role in enhancing performance. Most water soluble vitamins and vitamin E are involved in mitochondrial energy metabolism, yet the influence of vitamin supplementation on mitochondrial metabolism is largely unknown [21]. Vitamin E has a strong antioxidant capacity and has been used in several ischemia-reperfusion studies. It plays a major role in maintaining cell membrane integrity by limiting lipid peroxidation by ROS [22]. Moreover, vitamin E supplementation has been show to decrease renal injuries due to its antioxidant property [22-24]. On the other hand, no previous study demonstrated the effects of vitamin E supplementation on oxidative stress induced by exercise stress in just weaned rats. Our results are supported by the literature which strongly indicates vitamin E as a potent antioxidant.

Reduced glutathione has been reported as one of the major non-enzymatic antioxidants which detoxify peroxides/hydroperoxides. It has been demonstrated that intracellular redox status alterations are associated with depletion of reduced glutathione [25]. Based on our data, in association with reduced renal MDA levels, vitamin E supplementation increased renal reduced glutathione levels in rats submitted to exercise stress, which supports its antioxidant effect on rats submitted to exhaustive exercise stress. In addition, a previous study showed that vitamin E restored renal reduced glutathione levels and protected rat kidneys against oxidative stress induced by hyperthyroidism [26].

According to our results, the antioxidant effects of vitamin E supplementation on rat's kidney are supported by the increased levels of renal vitamin E in the animals submitted to exercise stress and treated with vitamin E. Vitamin C and E treatment presented positive effects on the kidney of growing pigs [27]. Furthermore, vitamin E supplementation was already shown to present benefic effects on renal non-enzymatic antioxidants, it maintained renal vitamin E and reduced glutathione levels in rats exposed to polychlorinated biphenyls [28].

In this study we focused the antioxidant effects of vitamin E on the kidney of rats submitted to exhaustive exercise stress. There are many studies which emphasize the effects of exercise stress on different organs. Aydin et al [29] suggested that long term dietary restriction protect against endurance and exhaustive swimming exercise-induced oxidative stress in rats brain by inhibiting oxidative stress. However, a study from the laboratory of Kayatekin [30] showed that a single bout of exhaustive exercise was not able to change antioxidant enzyme activities and lipid peroxidation levels in rat hippocampus, prefrontal cortex and striatum. Bachur et al [31] investigated the anti-oxidative systems in rat skeletal muscle after acute physical exercise and observed increased reactive oxygen species production in this tissue. We suggest futures studies to evaluate the effects of vitamin E treatment on the oxidative status in different organs.

The ability of vitamin E to provide non-enzymatic antioxidant protection makes it a potentially valuable tool in the treatment of dysfunctions resulting from increase reactive oxygen species production. As a consequence, the administration of vitamin E caused a significant increase in renal vitamin E and GSH and a significant reduction in renal MDA in experimental exhaustive stress.

Our results suggest the existence of a protective effect opposing the oxidative stress produced by swimming in animals treated with vitamin E. The decreased production of reactive oxygen species are reflected by the reduced levels of renal MDA. The increase in the levels of vitamin E and GSH in the kidney of the rats submitted to exhaustive exercise stress constitutes an adjustment response by activating the non-enzymatic antioxidant system.

### Clinical aspects

Previous studies already investigated the impact of supplements on exhaustive exercise stress [32,33]. Vitamin E administration in children with immunoglobulin A (IgA) nephropathy, focal segmental glomerulosclerosis (FSGS) and type I diabetes demonstrated potential towards ameliorating progression. Oral vitamin E treatment was shown to reduce endothelial dysfunction, lipid peroxidation and oxidative stress in patients with chronic kidney failure (CKF) [34]. Considering that more than 40% of people who practice exercise make use of dietetic supplementation and that vitamin E is one of the most consumed supplementations [8], these issues regarding the efficacy and safety of vitamin E in the kidney are important to strength the relevance of this vitamin as a supplement to physical active people. Our results are important to indicate vitamin E as a good supplement in cases of subjects exposed to exhaustive exercise, such as marathon and also for those exposed to a high intensity stress which required high energy consumption. Due to its antioxidant property, vitamin E presents renal protective effects and could be used as nutrient supplements in kidney disorders caused by exhaustive exercise.

### Limitations

Our investigation presents some points that should be addressed, we did not measure the concentration of hydrogen peroxide (H_2_O_2_), superoxide anions (SO^2-^) and NADPH oxidase subunities (p22^phox^, p40^phox^, p47^phox^, gp91^phox ^and p67^phox^) in the kidney. It would significantly strengthen the impact of our results. Unfortunately, we did not measure those components due to the lack of such equipment in our laboratory. However, we clearly showed that vitamin E supplementation is able to reduce renal oxidative stress induced by exercise stress through renal MDA measurement. MDA is a rather insensitive index of lipid oxidation. Nevertheless, previous studies confirmed its significant relationship with oxidative stress [35-38].

## Conclusions

Vitamin E supplementation improved renal non-enzymatic antioxidant activity in young rats submitted to exhaustive exercise stress. Our findings strength the importance of vitamin E supplementation in exercise stress situations.

## Abbreviations

LP: Lipid peroxidation; MDA: Malondialdehyde; GSH: Reduced glutathione; TBARS: Thiobarbituric acid reactive substance; HPLC: High-performance liquid chromatography; ROS: Reactive oxygen species; VO_2_: Oxygen consumption; XO: xanthine oxidase; IgA: immunoglobulin A; FSGS: focal segmental glomerulosclerosis; CKF: Chronic kidney failure; H_2_O_2_: Hydrogen peroxide; SO^2-^: Superoxide anions

## Competing interests

The authors declare that they have no competing interests.

## Authors' contributions

All authors participated in the acquisition of data and revision of the manuscript. SAB, LCA, VEV and HV conceived of the study, determined the design, performed the statistical analysis, interpreted the data and drafted the manuscript. All authors conceived of the study, determined the design, interpreted the data and drafted the manuscript. All authors read and gave final approval for the version submitted for publication.

## Pre-publication history

The pre-publication history for this paper can be accessed here:

http://www.biomedcentral.com/1472-6882/11/133/prepub

## References

[B1] Ji LL Antioxidants and oxidative stress in exercise Proc Soc Exp Biol Med 1999 222 283 292 10.1046/j.1525-1373.1999.d01-145.x 10601887

[B2] Ohishi S Kizaki T Ookawara T Toshinai K Haga S Karasawa F Satoh T Nagata N Ji LL Ohno H The effect of exhaustive exercise on the antioxidant enzyme system in skeletal muscle from calcium-deficient rats Pflugers Arch 1998 435 767 74 10.1007/s004240050582 9518504

[B3] Aydin C Sonat F Sahin SK Cangul IT Ozkaya G Long term dietary restriction ameliorates swimming exercise-induced oxidative stress in brain and lung of middle-aged rat Indian J Exp Biol 2009 47 24 31 19317348

[B4] Bright-Gbebry M Makambi KH Rohan JP Llanos AA Rosenberg L Palmer JR Adams-Campbell LL BMC Complement Altern Med 2011 11 30 10.1186/1472-6882-11-30 21496245 PMC3095573

[B5] Jakesevic M Aaby K Borge GI Jeppsson B Ahrné S Molin G Antioxidative protection of dietary bilberry, chokeberry and Lactobacillus plantarum HEAL19 in mice subjected to intestinal oxidative stress by ischemia-reperfusion BMC Complement Altern Med 2011 11 8 10.1186/1472-6882-11-8 21272305 PMC3038167

[B6] Shen CL Chyu MC Pence BC Yeh JK Zhang Y Felton CK Doctolero S Wang JS Green tea polyphenols supplementation and Tai Chi exercise for postmenopausal osteopenic women: safety and quality of life report BMC Complement Altern Med 2010 10 76 10.1186/1472-6882-10-76 21143878 PMC3014873

[B7] Leung J Larive B Dwyer J Hibberd P Jacques P Rand W HEMO Study Group Folic acid supplementation and cardiac and stroke mortality among hemodialysis patients J Ren Nutr 2010 20 293 302 10.1053/j.jrn.2010.01.005 20303789 PMC2892247

[B8] Halsted CH Dietary supplements and The American Journal of Clinical Nutrition Am J Clin Nutr 2000 71 399 400 10648249 10.1093/ajcn/71.2.399

[B9] Haidara MA Mikhailidis DP Rateb MA Ahmed ZA Yassin HZ Ibrahim IM Rashed LA Evaluation of the effect of oxidative stress and vitamin E supplementation on renal function in rats with streptozotocin-induced Type 1 diabetes J Diabetes Complications 2009 23 130 6 10.1016/j.jdiacomp.2008.02.011 18436458

[B10] Bucioli SA de Abreu LC Valenti VE Vannucchi H Carnitine suplementation effects on non-enzymatic antioxidants in young rats submitted to exhaustive exercise stress J Strength Cond Res 2011 in press 10.1519/JSC.0b013e318234ebcb 21912289

[B11] Schröder H Navarro E Mora J Galiano D Tramullas A Effects of alpha-tocopherol, beta-carotene and ascorbic acid on oxidative, hormonal and enzymatic exercise stress markers in habitual training activity of professional basketball players Eur J Nutr 2001 40 178 184 10.1007/s003940170006 11905959

[B12] Naziroğlu M Kilinç F Uğuz AC Celik O Bal R Butterworth PJ Baydar ML Oral vitamin C and E combination modulates blood lipid peroxidation and antioxidant vitamin levels in maximal exercising basketball players Cell Biochem Funct 2010 28 300 5 10.1002/cbf.1657 20517894

[B13] Naziroğlu M Şimşek M Kutlu M Moderate exercise with dietary vitamin C and E combination protects streptozotocin-induced oxidative damage to the blood and improves fetal outcomes in pregnant rats Clin Chem Lab Med 2004 42 511 517 10.1515/CCLM.2004.087 15202787

[B14] Mastaloudis A Traber MG Carstensen K Widrick JJ Antioxidants did not prevent muscle damage in response to an ultramarathon run Med Sci Sports Exerc 2006 38 72 80 16394956 10.1249/01.mss.0000188579.36272.f6

[B15] Cholewa J Poprzecki S Zalac A The influence of vitamin C on blood oxidative stress parameters in basketball players in response to maximal exercise Sci Sports 2008 23 176 182 10.1016/j.scispo.2008.01.004

[B16] Gündüz F Sentürk UK Kuru O Aktekin B Aktekin MR The effect of one year's swimming exercise on oxidant stress and antioxidant capacity in aged rats Physiol Res 2004 53 171 176 15046553

[B17] Radák Z Kaneko T Tahara S Nakamoto H Ohno H Sasvári M Nyakas C Goto S The effect of exercise training on oxidative damage of lipids, proteins, and DNA in rat skeletal muscle: evidence for beneficial outcomes Free Radic Biol Med 1999 27 69 74 10.1016/S0891-5849(99)00038-6 10443921

[B18] Uchiyama M Mihara M Determination of malonaldehyde precursor in tissues by thiobarbituric acid test Anal Biochem 1978 86 271 278 10.1016/0003-2697(78)90342-1 655387

[B19] Sedlak J Lindsay RH Estimation of total, proteinbound, and nonprotein sulfhydryl groups in tissue with Ellman's reagent Anal Biochem 1968 25 192 205 4973948 10.1016/0003-2697(68)90092-4

[B20] Arnaud J Fortis I Blachier S Kia D Favier A Simultaneous determination of retinol, a-tocopherol and b-carotene in serum by isocratic high-performance liquid chromatography J Chromatogr 1991 572 103 116 10.1016/0378-4347(91)80476-S 1818046

[B21] Ewans W Vitamin E. Vitamin C and exercise Am J Clin Nutr 2000 72 647 652

[B22] Canbaz S Duran E Ege T Sunar H Cikirikcioglu M Acipayam M The effect of intracoronary administration of vitamin E on myocardial ischemia-reperfusion injury during coronary artery surgery Thorac Cardiovasc Surg 2003 51 57 61 10.1055/s-2003-38983 12730811

[B23] Ibrahim W Lee US Szabo J Bruckner G Chow CK Oxidative stress and antioxidant status in mouse kidney: effects of dietary lipid and vitamin E plus iron J Nutr Biochem 1999 10 674 8 10.1016/S0955-2863(99)00053-4 15539266

[B24] Fryer MJ Vitamin E may slow kidney failure owing to oxidative stress Redox Rep 1997 3 259 61 9754323 10.1080/13510002.1997.11747121

[B25] Paker L Oxidants, antioxidants nutrients and the atletes J Sports Sci 1997 15 353 363 10.1080/026404197367362 9232561

[B26] Luck MR Jeyaseelan I Scholes RA Ascorbic acid and fertility Biol Reprod 1995 52 262 266 10.1095/biolreprod52.2.262 7711198

[B27] Jena S Chainy GB Regulation of expression of antioxidant enzymes by vitamin E and curcumin in L: -thyroxine-induced oxidative stress in rat renal cortex Mol Biol Rep 2010 in press 10.1007/s11033-010-0201-4 20574713

[B28] Eichenberger B Pfirter HP Wenk C Gebert S Influence of dietary vitamin E and C supplementation on vitamin E and C content and thiobarbituric acid reactive substances (TBARS) in different tissues of growing pigs Arch Anim Nutr 2004 58 195 208 10.1080/00039420410001701413 15264669

[B29] Aydin C Sonat F Sahin SK Cangul IT Ozkaya G Long term dietary restriction ameliorates swimming exercise-induced oxidative stress in brain and lung of middle-aged rat Indian J Exp Biol 2009 4 24 31 19317348

[B30] Acikgoz O Aksu I Topcu A Kayatekin BM Acute exhaustive exercise does not alter lipid peroxidation levels and antioxidant enzyme activities in rat hippocampus, prefrontal cortex and striatum Neurosci Lett 2006 406 148 51 10.1016/j.neulet.2006.07.034 16905254

[B31] Bachur JA Garcia SB Vannucchi H Jordao AA Chiarello PG Zucoloto S Anti-oxidative systems in rat skeletal muscle after acute physical exercise Appl Physiol Nutr Metab 2007 32 190 6 10.1139/h06-078 17486159

[B32] Paula FB Gouvêa CM Alfredo PP Salgado I Protective action of a hexane crude extract of Pterodon emarginatus fruits against oxidative and nitrosative stress induced by acute exercise in rats BMC Complement Altern Med 2005 5 17 10.1186/1472-6882-5-17 16107219 PMC1192789

[B33] Kayatekin BM Uysal N Resmi H Bediz CS Temiz-Artmann A Genç S Tugyan K Açikgöz O Gönenç S Akhisaroglu M Cehreli R Does antioxidant supplementation alter the effects of acute exercise on erythrocyte aggregation, deformability and endothelium adhesion in untrained rats? Int J Vitam Nutr Res 2005 75 243 50 10.1024/0300-9831.75.4.243 16229340

[B34] Thabet MA Chan JC Vitamin E in renal therapeutic regiments Pediatr Nephrol 2006 21 1790 801 10.1007/s00467-006-0211-6 17186590

[B35] Ciocoiu M Badescu M Paduraru I Protecting antioxidative effects of vitamins E and C in experimental physical stress J Physiol Biochem 2007 63 187 94 10.1007/BF03165781 18309774

[B36] Ciocoiu M Badescu MM Lupusoru EC The intervention of antioxidant therapy on platelet adhesion and immunomodulation in experimental physical stress Free Radic Res 2007 41 829 38 10.1080/10715760701416434 17577744

[B37] Jakesevic M Aaby K Borge GI Jeppsson B Ahrné S Molin G Antioxidative protection of dietary bilberry, chokeberry and Lactobacillus plantarum HEAL19 in mice subjected to intestinal oxidative stress by ischemia-reperfusion BMC Complement Altern Med 2011 11 8 10.1186/1472-6882-11-8 21272305 PMC3038167

[B38] Gordon LA Morrison EY McGrowder DA Young R Fraser YT Zamora EM Alexander-Lindo RL Irving RR Effect of exercise therapy on lipid profile and oxidative stress indicators in patients with type 2 diabetes BMC Complement Altern Med 2008 8 21 10.1186/1472-6882-8-21 18477407 PMC2390515

